# The Fermentation Stress Response Protein Aaf1p/Yml081Wp Regulates Acetate Production in *Saccharomyces cerevisiae*

**DOI:** 10.1371/journal.pone.0051551

**Published:** 2012-12-11

**Authors:** Christopher J. Walkey, Zongli Luo, Lufiani L. Madilao, Hennie J. J. van Vuuren

**Affiliations:** Wine Research Centre, University of British Columbia, Vancouver, British Columbia, Canada; Cankiri Karatekin University, Turkey

## Abstract

The production of acetic acid during wine fermentation is a critical issue for wineries since the sensory quality of a wine can be affected by the amount of acetic acid it contains. We found that the C2H2-type zinc-finger transcription factor YML081Wp regulated the mRNA levels of *ALD4 and ALD6*, which encode a cytosolic acetaldehyde dehydrogenase (ACDH) and a mitochondrial ACDH, respectively. These enzymes produce acetate from acetaldehyde as part of the pyruvate dehydrogenase bypass. This regulation was also reflected in the protein levels of Ald4p and Ald6p, as well as total ACDH activity. In the absence of *ALD6*, YML081W had no effect on acetic acid levels, suggesting that this transcription factor’s effects are mediated primarily through this gene. *lacZ* reporter assays revealed that Yml081wp stimulates *ALD6* transcription, in large part from a GAGGGG element 590 base pairs upstream of the translation start site. The non-annotated ORF YML081W therefore encodes a transcription factor that regulates acetate production in *Saccharomyces cerevisiae.* We propose *AAF1* as a gene name for the YML081W ORF.

## Introduction

During fermentation, yeast cells are exposed to a challenging environment: low pH, hypoxia, high osmotic pressure, and a rising ethanol concentration. The yeast cells respond to these stresses by activating a gene expression program called the Fermentation Stress Response (FSR) [1]. We previously found that mRNA levels for 224 genes were elevated in a significant and sustained manner. Interestingly, 62 of these genes are non-annotated; they are not linked to any specific function. Therefore, we have been exploring wine fermentation as a means for elucidating a role for these genes in yeast metabolism.

We created wine yeast strains that carry null mutations for each of the 62 non-annotated FSR genes. As well, we created strains that overexpress each non-annotated FSR gene. These strains were used to perform wine fermentations. We observed that the null strain for one FSR gene, YML081W, produced less acetic acid than its wild-type counterpart. The strain overexpressing YML081W produced wine with elevated acetic acid levels. Yml081wp is classified as a putative transcription factor, containing a C2H2-like zinc finger domain at the N-terminus. However, its biological role is largely unknown; it has only been linked to resistance to topoisomerase I-induced DNA damage [2]. Therefore, we investigated the role and mechanism of Yml081Wp in regulating acetic acid production.

The primary biosynthetic pathway for acetate is the pyruvate dehydrogenase bypass [3]. In the cytoplasm, pyruvate is decarboxylated to acetaldehyde, a portion of which is subsequently oxidized to acetate, with the concomitant reduction of NADP^+^ to NADPH. Acetate is the sole source of acetyl-CoA in the cytoplasm, which is a crucial precursor for anabolic processes in the yeast cell. Excess acetate is excreted from the cell as acetic acid. *ALD6* encodes the primary cytosolic acetaldehyde dehydrogenase (ACDH) activity. Cells lacking *ALD6* are defective in growth [4], and produce less acetic acid during fermentation [5], [6]. *ALD2* and *ALD3* also encode cytosolic aldehyde dehydrogenases, but these genes do not play a role in acetic acid production [6], [7].

Acetaldehyde can also be oxidized to acetate in mitochondria by two different ACDH enzymes, encoded by *ALD4* and *ALD5*. Deletion studies suggest that these enzymes are not as important as *ALD6* for the production of acetic acid, although cells lacking *ALD5* did produce slightly less acetic acid during wine fermentation [6].

Given our preliminary data linking YML081W to extracellular acetic acid levels, we investigated this transcription factor’s regulation of intracellular acetate production. This paper shows that Yml081wp stimulates expression of both the *ALD4* and *ALD6* genes, and that the regulation of *ALD6* in particular is the basis of its acetic acid phenotype. We propose naming this gene *AAF1*, for Acetic Acid Factor 1.

## Materials and Methods

### Strain Construction

All strains used in this paper were based on the industrial M2 wine yeast [8], [9]. This strain of *Saccharomyces cerevisiae* was chosen for its amenability to genetic manipulation, as well as its ability to carry out industrial wine fermentations. DNA cassettes designed to alter specific genes were generated by PCR, using the oligonucleotides listed in Table S1. For YML081W-null and *RSF2*-null strains, a *loxP-kanMX4-loxP* cassette was amplified from the plasmid pUG6 [10]. For *ALD4*-null and *ALD6*-null strains, an *hphMX4* cassette was amplified from the plasmid pAG32 [11]. The PCR primers contained 17–19 nucleotides at the 3′ end designed to amplify the cassette, and 60–70 nucleotides at the 5′ends identical to the flanking sequences of each targeted open reading frame (ORF). iProof kits (Bio-Rad) were used for PCR amplification. The amplicons were introduced into the target cells by standard lithium acetate transformation. Following recombination, each target gene’s ORF was replaced by the *loxP-kanMX4-loxP* or *hphMX4* cassette. Transformants were selected by antibiotic resistance, and the correct DNA recombination confirmed by colony PCR. Since the M2 strain is diploid, the transformed cells were sporulated, and the resulting tetrads dissected. The haploid colonies, which spontaneously revert to diploid, were selected for antibiotic resistance, thus generating strains that were homozygous for the intended mutation. The absence of the target gene was confirmed by colony PCR.

In order to generate strains overexpressing our genes of interest, the pCW1 plasmid containing the *PGK1* promoter was constructed. Briefly, 788 bp upstream of the *PGK1* gene were amplified from the pHVX2 vector [12], and cloned into the Sal1 site of pUG6. InFusion HD kits (Clontech) were used for cloning. The resulting plasmid was used as a template to generate the amplicons containing the *PGK1* promoter linked the *kanMX4* selection cassette. Transformations were carried out as above.

Table S2 contains the complete list of yeast strains.

### Wine Fermentations

Wine fermentations were carried out as described previously [13], using 70 ml filter-sterilized Calona Chardonnay grape juice at 19°C, in triplicate. At the completion of fermentation, the major wine components (glucose, fructose, glycerol, acetic acid and ethanol) were assayed by HPLC, with a 5 µl injection volume [14].

### Aerobic Growth

Yeast cells were also grown aerobically at 30°C, in triplicate. Typically, the cells were grown to mid-log phase (OD_600_ = 1.5–2.0) in YPD media (1% yeast extract, 2% peptone, 2% dextrose). Media components (except fructose) were assayed as above by HPLC. Injection volumes were doubled to 10 µl, to compensate for the lower concentrations of these components in lab media compared to wine.

### Quantitative PCR

Total RNA was isolated from mid-log phase cells using a yeast-specific RiboPure kit (Ambion); 1 µg of total RNA was converted to cDNA using the Vilo reverse transcription kit (Invitrogen). mRNA levels were determined by PCR on an Applied Biosystems 7500 Real Time PCR System, using SYBR Green, and calculating ΔΔC_t_. The primers used for amplification are listed in Table S1. All primers were validated for linear amplification. mRNA levels in each strain were determined from three separate cultures, and resulting sample was amplified in triplicate. Amplification of *TAF10* cDNA was used for normalization.

### Immunoblotting

Yeast cells were grown to mid-log phase, and crude lysates prepared in SDS loading buffer as previously described [13]. Lysates corresponding to ∼0.1 OD_600_ units of cells were electrophoresed on a 4–15% gradient gel, and then transferred to nitrocellulose membrane. The membrane was stained (MemCode, Pierce) in order to confirm equal loading. Immunoblotting was carried out according to standard protocols. The primary antibody was α-FLAG (M2 variant, Sigma), diluted 10000-fold.

### ACDH Assay

Acetaldehyde dehydrogenase activity was assayed in whole cell lysates, as previously described [6], [15]. Each strain was assayed in triplicate from separate cultures. Approximately 1 OD_600_ unit of mid-log phase yeast cells was pelleted by brief microcentrifugation, and then washed twice in 10 mM potassium phosphate buffer, pH 7.5. The washed cells were resuspended in 100 µl of lysis buffer (100 mM potassium phosphate buffer, pH 7.5, 2 mM MgCl_2_, 1 mM dithiothreitol), and lysed by mechanical shearing. The cell debris was removed by microcentrifugation.

The ACDH assay was performed by adding 10 µl of lysate to 1 ml of assay buffer (100 mM potassium phosphate, pH 8.0, 15 mM pyrazole, 10 mM MgCl_2_, 0.4 mM NAD^+^, 0.4 mM NADP^+^, 0.4 mM dithiothreitol). Following pre-incubation at 30°C, the reaction was started by addition of acetaldehyde to a final concentration of 0.3 mM. The progress of the reaction was followed by measuring the increase in A_340_ at 10 minute intervals for 2 hours.

The total protein content of the cell lysates was measured using the BCA protein assay kit according to the manufacturer’s (Pierce) instructions. The final specific enzyme activity was calculated from the rate of change of A_340_, and converted to NAD(P)H concentration with an extinction coefficient of 6220. The units are [nmol NAD(P)H formed/min]/mg protein.

### lacZ Reporter Assays

Because our experiments were performed in an industrial wine strain without auxotrophic selection, it was necessary to construct a *lacZ* translational fusion reporter plasmid that encoded a marker for positive selection. Using the pMELβ2 plasmid as a template [16], the *lacZ* cassette was amplified by PCR, and cloned into the SalI site of pYC140 [17]. The PCR primers were designed to maintain the SalI site at the 5′ end of the *lacZ* sequence for subsequent cloning of promoter fragments, but abolished the SalI site at the 3′ end. The resulting plasmid, labeled pCW5, contained a hygromycin resistance cassette for selection, and the CEN/ARS sequence for replication.

In order to construct *ALD6* promoter reporters, the upstream region of the *ALD6* gene, plus the first 22 nucleotides of the coding region, was amplified by PCR, using purified M2 strain genomic DNA as a template. A series of reporters was created, covering different lengths of the *ALD6* upstream region (−782, −636, −586, −520 and −414). These fragments were cloned into the SalI site of pCW5 to generate *ALD6* promoter-*lacZ* reporters. The resulting plasmids were sequenced to confirm the fidelity of the PCR reaction.

The *lacZ* reporter plasmids were transformed into the M2 wine strains, both wild-type and *yml081W*Δ::*KanMX4*, using the LiOAc method. Transformants were selected by growth on YPD plates containing 300 µg/ml hygromycin B (Invitrogen). For each experiment, three independent transformants for each strain and plasmid were chosen and grown to mid-log phase in YPD/hygromycin. Cell density was measured by OD_600_, and each sample was assayed for β-galactosidase activity with a kit (Pierce), using 100 µl of culture in a final volume of 0.3 ml. Yellow colour development after a 30-minute incubation at 37°C was measured by absorbance at 420 nm. β-galactosidase activity was calculated in Miller units from the formula activity = A_420_×1000/(OD_600_×30 min×0.3 ml).

In order to create mutations of potential YML081W binding sites in the *ALD6* promoter, site-directed mutagenesis of the *ALD6* promoter *lacZ* reporters was performed using the Quikchange II kit (Stratagene). The resulting plasmids were sequenced to ensure the presence of the expected mutation.

## Results

### Yml081Wp Controls Acetic Acid Levels

**Figure 1:**
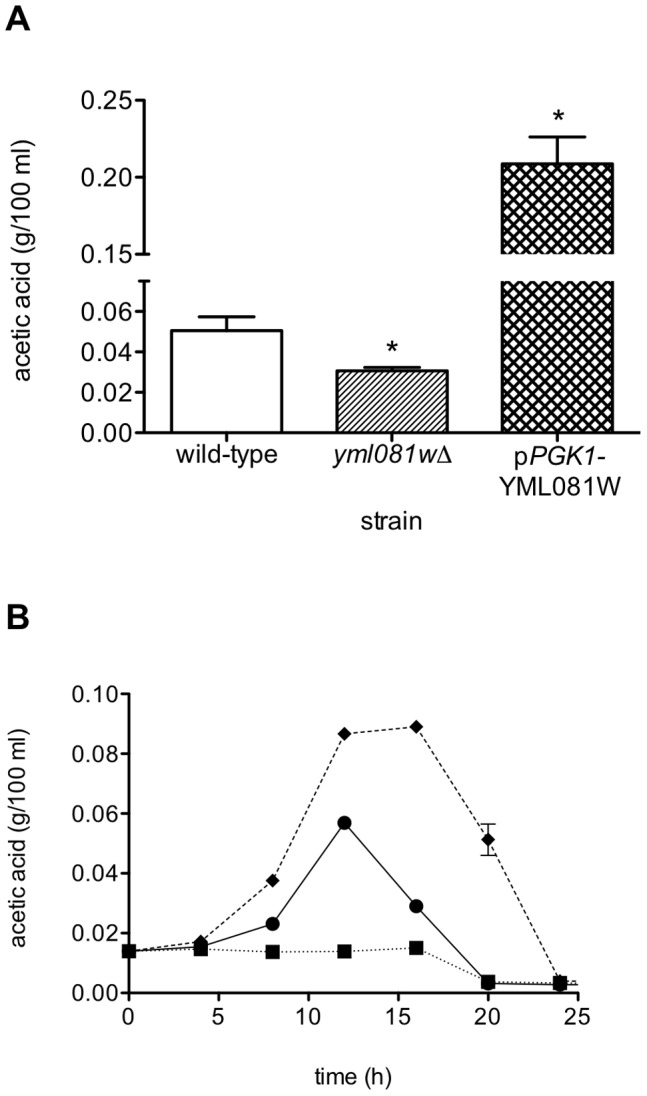
Yml081Wp regulates acetic acid levels. (**A**) Wild-type, YML081W-null and YML081W-overexpressing M2 yeast cells were used in a wine fermentation with sterile Chardonnay grape juice. Once fermentation was complete, the wine was assayed for acetic acid by HPLC. YML081W-null cells produced significantly less acetic acid, while YML081W-overexpressing cells produced significantly more acetic acid. In this figure, and all subsequent figures, * indicates p<0.05 for a two-tailed Student t-test, compared to wild-type. (**B**) These same strains were grown in triplicate in YPD media. At 4 hour intervals, media samples were removed, and assayed for acetic acid as above. YML081-null cells (▪, dotted line) produced significantly less acetic acid in the media than wild-type cells (•, solid line), while YML081W-overexpressing cells (⧫, dashed line) produced significantly more acetic acid.

Two modified *S. cerevisiae* M2 wine yeast strains were constructed: (1) a YML081W-null strain, and (2) a YML081W-overexpression strain. These strains were used to perform wine fermentations in Chardonnay grape juice, which contains negligible amounts of acetic acid. At the completion of fermentation after 21 days, the wine was assayed for acetic acid, by HPLC. The YML081W-null strain produced 39.1% less acetic acid in the wine than the wild-type strain (Figure 1A). The strain over-expressing YML081W produced 4.14-fold more acetic acid than its wild-type counterpart. These results showed a positive correlation between YML081W and acetic acid levels.

To further investigate this phenotype, we examined the role of YML081W in acetic acid production during standard lab growth. YML081W-null and YML081W –overexpressing strains, along with the wild-type M2 control strain, were grown in YPD media. Media samples were removed periodically, and assayed for acetic acid. As shown in Figure 1B, yeast cells lacking YML081W produced significantly less acetic acid than wild-type yeast cells during the first 12 hours of growth. Conversely, yeast cells overexpressing YML081W produced significantly more acetic acid. After 12–16 hours of growth, the diauxic shift occurred, and the cells began consuming acetic acid from the media. These results in YPD media are similar to those seen during wine fermentation. Subsequent experiments were performed with these strains during YPD growth, since the effects were detectable within hours, rather than the weeks-long time frame of wine fermentation.

**Figure 2:**
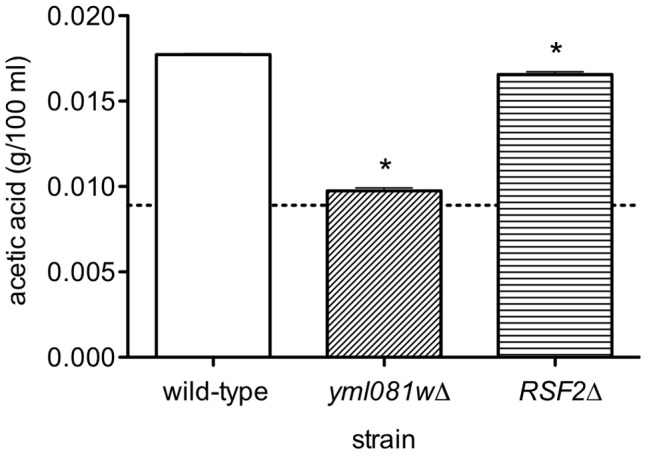
The acetic acid effect is much stronger with YML081W than with its ohnolog *RSF2*. Wild-type, YML081W-null and *RSF2*-null strains were grown in triplicate to mid-log phase in YPD media, and the resulting media was assayed for acetic acid. The dashed line indicates the initial acetic acid level of the media. Elimination of YML081W has a much more dramatic effect on acetic acid levels than elimination of *RSF2*.

Yml081Wp is closely related to another transcription factor, Rsf2p. This pairing most likely arose from whole genome duplication, and is hence considered ohnologous. Their sequences share 38.0% identity and 54.5% similarity. Within the zinc-finger domains near the N-terminus of each protein, the sequences are 80.0% identical. Previous studies have indirectly implicated *RSF2* in the regulation of acetic acid levels [18]. Therefore, we constructed an M2-based *RSF2*-null strain, and tested it for its ability to produce acetic acid. As shown in Figure 2, *RSF2*-null cells produced slightly less acetic acid when grown in YPD, compared to wild-type cells. However, the effect of deleting YML081W was significantly stronger. Therefore, we consider YML081W to be much more important for regulating acetic acid levels than its ohnolog *RSF2*.

### Yml081Wp Regulates Expression of ALD4 and ALD6 Genes

**Figure 3:**
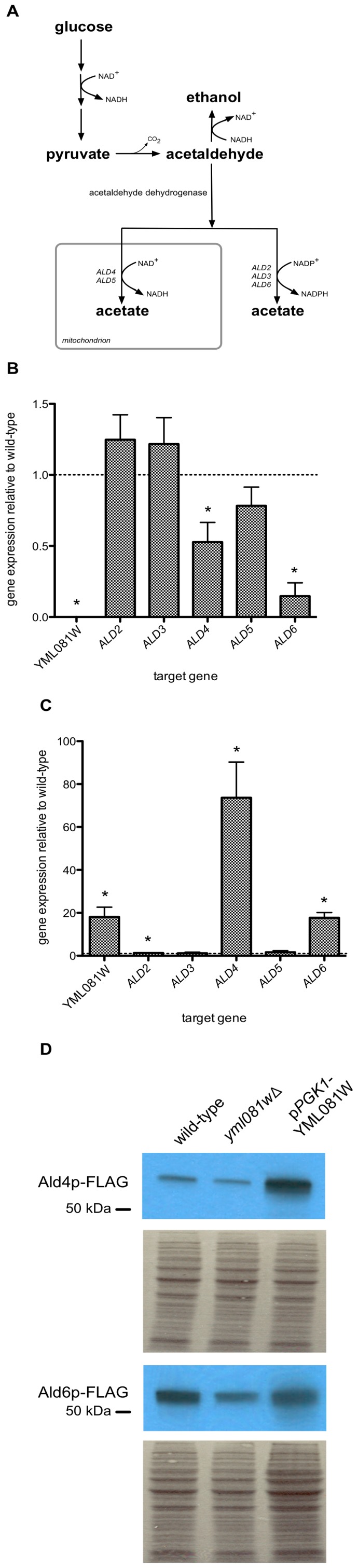
Yml081Wp regulates *ALD* gene expression and protein levels. (**A**) Acetate biosynthesis pathway, adapted from Saint-Prix *et al.* [6]. (**B**) *ALD* gene expression was measured in mid-log phase wild-type and YML081W-null cells by quantitative PCR. Removal of YML081W resulted in a significant reduction in *ALD4* and *ALD6* mRNA levels. (**C**) *ALD* gene expression was measured in mid-log phase yeast cells overexpressing YML081W under the control of the *PGK1* promoter, and compared to wild-type cells. YML081W-overexpressing cells produced significantly higher levels of ALD4 and ALD6 mRNA compared to wild-type cells. (**D**) *ALD4* and *ALD6* genes were tagged with the FLAG epitope at the C-terminus in wild-type, YML081W-null and YML081Woverexpressing strains. The cells were grown to mid-log phase, then harvested and lysed. The lysates were immunoblotted with an α-FLAG antibody to detect the levels of Ald4p-FLAG and Ald6p-FLAG protein. Cells lacking YML081W produced lower levels of FLAG-tagged Ald4p and Ald6p, compared to wild-type cells. Cells overexpressing YML081W produced significantly higher levels of Ald4p-FLAG protein compared to wild-type cells. ALD6p-FLAG levels did not appear to change significantly. Membrane staining shown below the immunoblots indicates the equivalance of total protein loading between lanes.

The primary pathway for intracellular acetate biosynthesis occurs through the oxidation of acetaldehyde, catalyzed by the enzyme acetaldehyde dehydrogenase (ACDH) (Figure 3A). In yeast, five genes, *ALD2-6*, have been identified as encoding ACDH activity [7]. However, *ALD4* and especially *ALD6* have been identified as the major genes responsible for acetate biosynthesis. As YML081W encodes a potential transcription factor, its role on the acetic acid production might be through its regulation on the transcription of the *ALD* genes. Therefore, we tested the effects of altering YML081W expression on the expression of *ALD* genes. Using quantitative PCR, we found that deletion of YML081W resulted in significant reductions in the mRNA of *ALD4* and *ALD6* (Figure 3B). Gene expression of *ALD4* was reduced 47.3% compared to wild-type, while gene expression of *ALD6* was reduced 85.3%. The mRNA levels of *ALD2*, *ALD3* and *ALD5* were not significantly altered by YML081W deletion. We also tested the effect of YML081W overexpression on *ALD* gene expression. Insertion of the highly active *PGK1* promoter resulted in an 18.1-fold increase in the YML081W mRNA level (Figure 3C), *ALD4* and *ALD6* gene expression were also dramatically increased. The *ALD4* mRNA level was boosted 73.6-fold, while the *ALD6* mRNA level was increased 17.7-fold. *ALD2* showed a very small (1.25-fold), but statistically significant increase in gene expression. *ALD3* and *ALD5* mRNA levels were not affected by YML081W overexpression.

**Figure 4:**
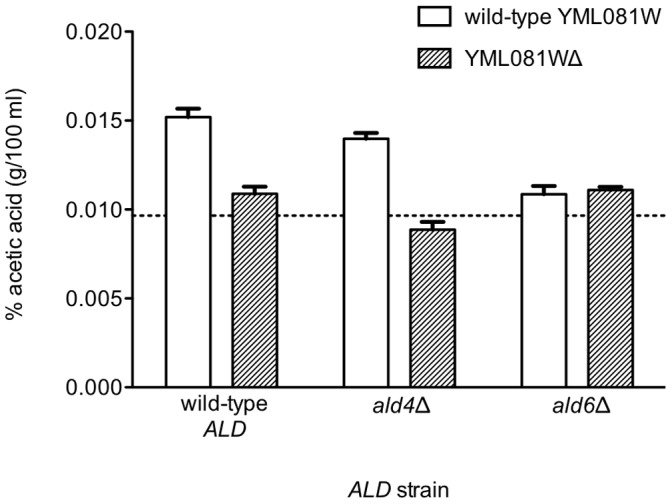
The Yml081Wp effect on acetate production requires *ALD6*. *ALD4*-null and *ALD6*-null M2 yeast strains with and without YML081W were grown to mid-log phase, and then assayed for acetic acid production by HPLC. The dashed line indicates the initial acetic acid level of the media. As expected, with the full complement of *ALD* genes, eliminating YML081W produced a significant reduction in acetic acid levels (compare the two leftmost columns). Eliminating *ALD6* produced a lower acetic acid level compared to wild-type (compare the first and fifth columns). However, the further elimination of YML081W had no effect (compare the fifth and sixth columns). Therefore, YML081W requires *ALD6* to mediate its effects on acetic acid levels.

Next, we tested whether these changes at the mRNA level were reflected at the protein level. We constructed YML081W wild-type, null, and overexpression strains with the FLAG epitope linked to the C-terminus of the *ALD4* and *ALD6* genes. By immunoblotting with a commercially available α-FLAG antibody, we measured the effects of changing Yml081Wp levels on Ald4p and Ald6p expression. Deletion of YML081W resulted in lower levels of FLAG-tagged Ald4p and Ald6p (Figure 3D). Conversely, overexpression of YML081W resulted in a significant increase in the expression of Ald4p-FLAG. Interestingly, although *ALD6* mRNA was significantly increased by YML081W overexpression, Ald6p-FLAG levels were unaffected, suggesting a dysregulation between mRNA and protein levels under these particular conditions.

Therefore, given the clear positive correlation between YML081W and *ALD* genes, we examined whether YML081W affected the total acetaldehyde dehydrogenase activity in the yeast cells. ACDH was assayed in the presence of K^+^, Mg^++^, NAD^+^ and NAD(P)^+^, to avoid discriminating against any particular isoform. As shown in Table 1, deletion of YML081W resulted in a 59.0% reduction in ACDH activity. Overexpression of YML081W produced a 3.24-fold increase in ACDH activity. Therefore, the changes in *ALD* gene and protein levels produced by altering Yml081wp levels were reflected in the final enzyme activity.

**Table 1:** Yml081Wp regulates acetaldehyde dehydrogenase activity.

Strain	ACDH activity
wild-type	77.0±19.2
*yml081WΔ*	31.6±6.9
p*PGK1*-YML081W	249.7±53.3

M2 yeast cells were grown to mid-log phase, then harvested and lysed. ACDH specific activity was assayed as described in the Materials and Methods section. The numbers represent (nmol NAD(P)H formed/min) per mg protein. Cells without Yml081Wp contained lower ACDH activity, while cells overexpressing YML081W contained higher ACDH activity. These differences were statistically significant (p<0.05).

### ALD6 is Required for the Acetic Acid Phenotype of the YML081W Deletion Mutant

The previous results clearly showed a relationship between YML081W and acetaldehyde dehydrogenase gene expression and activity. Therefore, we wished to confirm whether this correlation was responsible for YML081W’s acetic acid phenotype. We constructed strains lacking either *ALD4* or *ALD6*, in the presence or absence of Yml081Wp. Cells with the full complement of *ALD* genes produced 77.7% less acetic acid (after correcting for the baseline acetic acid in the media) when YML081W was eliminated (Figure 4). Cells lacking *ALD6* produce 78.2% less acetic acid than their wild-type counterparts, demonstrating the importance of this gene for acetate production. However, in the absence of *ALD6*, the further elimination of YML081W produced no significant changes in acetic acid levels. Therefore, YML081W requires the presence of *ALD6* in order to regulate acetic acid levels.

*ALD4* had a smaller effect on acetic acid levels. With wild-type YML081W levels, *ALD4*-null cells produced 21.9% less acetic acid. When YML081W was deleted in an *ALD4*-null background, there was an additional 63.1% reduction in acetic acid levels. These results suggest that while YML081W regulates both *ALD4* and *ALD6*, this transcription factor’s acetic acid effects are mediated primarily through *ALD6*.

### Yml081Wp Stimulates ALD6 Transcription

**Figure 5:**
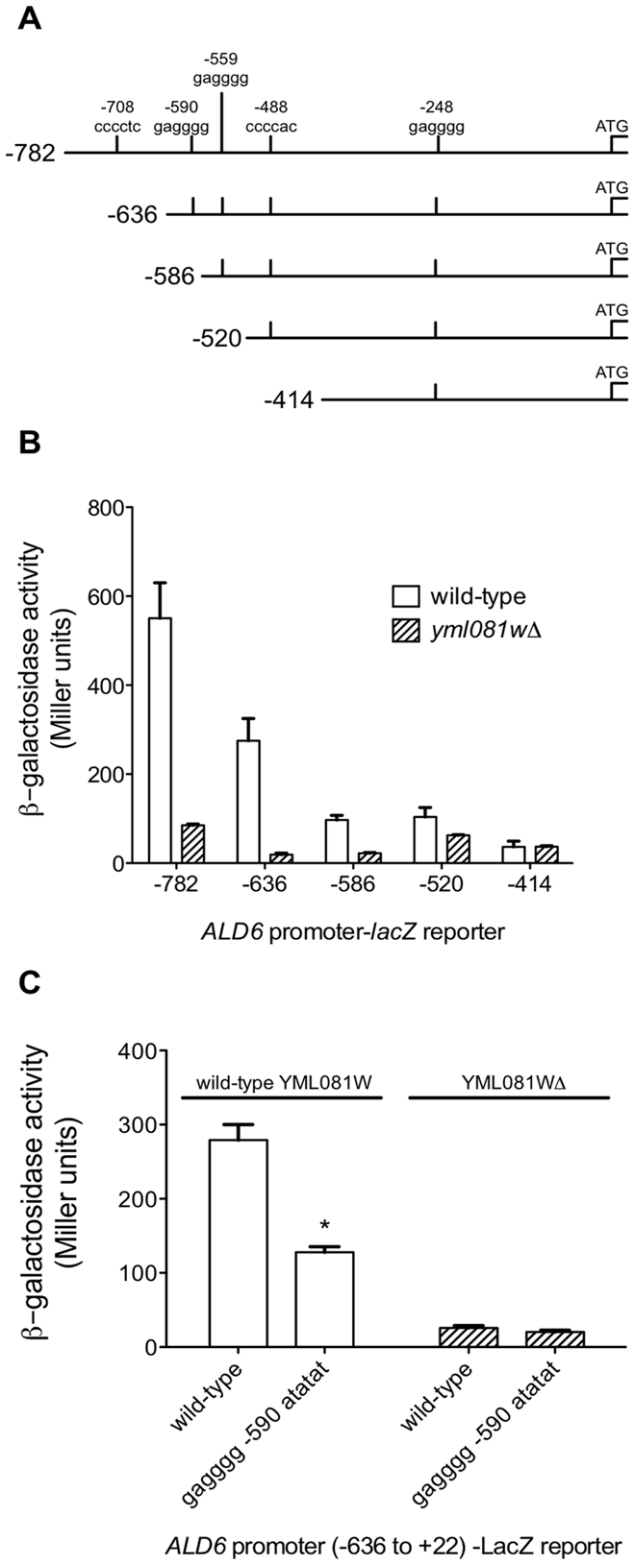
Yml081Wp regulates the *ALD6* promoter. (**A**) A schematic diagram of the *ALD6* promoter sequence. Numbers on the left indicate the position of the first nucleotide in the promoter construct relative to the start codon. The positions and sequences of sites matching the previously published Yml081Wp binding sites are highlighted above the full-length promoter. (**B**) LacZ reporter activity from the *ALD6* promoter. Reporter plasmids carrying *ALD6* promoter fragments of the indicated lengths were transformed into wild-type M2 and YML081W-null strains. Three independent transformants for each reporter were grown to log phase, and assayed for β-galactosidase activity. Truncation of the reporter resulted in progressively lower activity levels. Importantly, cells lacking Yml081Wp produced significantly lower β-galactosidase activity than their wild-type counterparts (except for the shortest reporter fragment), suggesting that this transcription factor plays a positive role in stimulating *ALD6* transcription. (**C**) A consensus binding site at −590 plays an important role in mediating Yml081Wp transcriptional activity on the *ALD6* promoter. A reporter plasmid carrying a mutation of the YML081W consensus binding site was constructed for comparison to its wild-type counterpart. In cells producing normal levels of Yml081Wp, the mutation resulted in a 54% reduction in β-galactosidase activity (* indicates p<0.05 for a two-tailed Student t-test, compared to wild-type). However, in cells lacking Yml081Wp, the mutation had no significant effect on β-galactosidase activity. This result suggests that the consensus binding site at position −590 mediates Yml081Wp transcriptional activity on the *ALD6* promoter.

Based on its primary sequence, Yml081Wp is classified as a C2H2-type zinc finger transcription factor. Its localization is strongly nuclear [19]. Therefore, we tested whether Yml081Wp stimulates transcription from the *ALD6* promoter. We constructed *lacZ* reporter plasmids based on the *ALD6* promoter (Figure 5A). When transformed into wild-type M2 cells, the plasmid containing the full-length *ALD6* promoter from 782 nucleotides upstream of the translation start site to 22 nucleotides downstream produced a significant β-galactosidase activity, indicative of transcriptional activity. However, in cells lacking YML081W, the same reporter plasmid produced 85% less β-galactosidase activity (Figure 5B). Plasmids without the *ALD6* promoter produced no detectable β-galactosidase activity (not shown). Therefore, the activity of the *ALD6* promoter is strongly dependent on the presence of Yml081Wp.

Global screens based on protein binding microarrays have identified the motif CCCCNC as a preferred DNA binding site for Yml081Wp [20]. The *ALD6* promoter contains five instances of this motif at the positions indicated in Figure 5A, in both orientations. To determine if one or more of these motifs are necessary for Yml081Wp activity, we constructed a series of truncated *ALD6* promoter reporters, designed specifically to eliminate one of these motifs at a time. In wild-type M2 cells, truncations that eliminated the motifs at positions −708, −590 and −488 produced significant reductions in β-galactosidase activity (Figure 5B). However, in YML081W-null cells, these truncations produced reductions in β-galactosidase activity of similar relative magnitude. The sole exception was the truncation that eliminated the segment between −636 and −586, where there is no difference in β-galactosidase activity in YML081W-null cells. This result suggested that the 60% drop in transcriptional activity between cells carrying the reporter plasmid with the −636 fragment of the *ALD6* promoter, and cells carrying the reporter plasmid with the −586 fragment of the *ALD6* promoter, is YML081W-dependent.

The segment of the *ALD6* promoter between −636 and −586 contains one putative Yml081Wp binding site: GAGGGG centered at position −590. To determine if this site is required for Yml081Wp-induced transcriptional activity, we constructed a mutated version of the *ALD6* promoter-*lacZ* reporter where this site was changed to atatat. In wild-type M2 cells, this mutation produced a 54% reduction in β-galactosidase activity (Figure 5C). In YML081W-null cells, the mutation had no significant effect on β-galactosidase activity. Therefore, this site required for Yml081Wp to fully stimulate transcription from the *ALD6* promoter.

## Discussion

During ethanolic yeast fermentation, pyruvate from glycolysis is decarboxylated to acetaldehyde. Most of this acetaldehyde is reduced to ethanol. However, a small portion is oxidized to acetate. This acetate serves as a precursor for acetyl-CoA, an important building block for anabolic metabolism, particularly lipid biosynthesis. This pathway, called the pyruvate dehydrogenase bypass, is critical for yeast, since it is the only source for acetyl-CoA in the cytoplasm [3], [7], [21]. Unused acetate diffuses into the media as acetic acid. Pyruvate that enters the mitochondria is also converted to acetyl-CoA by pyruvate dehydrogenase or by the mitochondrial pyruvate dehydrogenase bypass, but this acetyl-CoA is inaccessible to the rest of the cell.

Three yeast genes, *ALD2, ALD3* and *ALD6,* encode cytosolic aldehyde dehydrogenases. However, studies with *ALD*-null strains have conclusively shown that Ald6p is the primary acetaldehyde dehydrogenase (ACDH) in the yeast cell responsible for the oxidation of acetaldehyde to acetate [5], [6]. This enzyme is Mg^++^- and NAD(P)^+^-dependent [4], [22]. The precise roles for Ald2p and Ald3p are unclear. Null mutations of *ALD2* and *ALD3* have little effect on acetic acid levels during fermentation [6], [7]. More recent evidence suggests that they may play a role in beta-alanine synthesis [23].

We found that Yml081Wp is a critical regulator of *ALD6* expression, and hence acetic acid levels. Yeast cells that lack Yml081Wp produce significantly less acetic acid, both during standard lab growth, and during wine fermentation. These cells contain reduced amounts of *ALD6* mRNA and protein than their wild-type counterparts, and lower levels of ACDH activity. Interestingly, overexpression of YML081W boosted levels of *ALD6* mRNA, but not protein. In the absence of *ALD6*, deletion of YML081W had no effect on acetic acid levels during lab growth. Therefore, we concluded that Yml081Wp mediates its effect on acetic acid primarily through regulation of *ALD6*.

Since Yml081Wp is a C2H2-type zinc-finger transcription factor, we investigated whether it mediates its effects on acetic acid levels by directly stimulating transcription from the *ALD6* promoter. This class of transcription factors, the largest in yeast, typically binds as monomers to target promoters [24]. Using a custom-designed *lacZ* reporter plasmid, we found that the expression of Yml081Wp is necessary for full *ALD6* promoter expression. Therefore, we began searching for elements with the *ALD6* promoter that could be necessary for YML081W activity. We initially focused on a putative binding site, CACCCC, identified in a broad transcription factor binding survey using protein-binding microarrays. Using a series of reporter truncations and site-directed mutagenesis, we identified a site containing this sequence in reverse 590 base pairs upstream of the *ALD6* start codon that was required for the full activity of YML081W on this promoter. Interestingly, mutation of this site did not completely abolish Yml081Wp-mediated transcription, suggesting the presence of multiple Yml081Wp sites on the *ALD6* promoter. In a global scan of transcription factor binding sites by chromatin immunoprecipitation and genomic microarray analysis, *ALD6* was not identified as a Yml081Wp target [25]. However, a dubious ORF, YPL062W, which spans a region from 290 to 694 base pairs upstream of the *ALD6* start site, was identified as being proximal to a Yml081Wp binding site. This result is further evidence of a direct interaction between Yml081Wp and *ALD6*.

Yeast mitochondria also contain two ACDH isoforms, encoded by *ALD4* and *ALD5*. Ald4p uses both NAD^+^ and NADP^+^ as co-factors, and is K^+^-dependent [26]. Previous studies have shown that deletion of *ALD4* has little effect on acetate production, except in the absence of *ALD6*, where it appears to play a compensatory role [5]. Ald5p is also K^+^-dependent but only uses NADP^+^ as a co-factor. *ALD5* encodes only 20% of the total mitochondrial ACDH activity [27]. However, during wine fermentation, deletion of *ALD5* had a significantly larger effect on acetic acid levels than deletion of *ALD4* [6].

We found that deletion of YML081W specifically reduced the mRNA level of *ALD4*, though not to the same extent as for *ALD6*. The level of the FLAG-tagged Ald4p isoform was also reduced. Conversely, the overexpression of YML081W dramatically increased the levels of *ALD4* mRNA and Ald4p protein (unlike Ald6p). Most likely, the elevated Ald4p level was responsible for the higher ACDH activity in YML081W-overexpressing yeast. However, in the absence of *ALD4*, deletion of YML081W still reduced acetic acid levels, suggesting that this transcription factor’s regulation of *ALD4* is not its primary route for controlling acetic acid levels.

Control of volatile acidity (VA) is a critical issue for the industrial use of yeast. During wine fermentation, the production of acetic acid, the most abundant volatile acid, can have a dramatic effect on the quality of the final product. At levels typically found in wine, 0.02–0.06% (g/100 ml), acetic acid adds a pleasant tartness. Also, it serves as a precursor to acetate esters, which are responsible for the fruity character of many wines. However, acetic acid in wine is generally considered to be a spoilage product; acetic acid production can result in the formation of other unpleasant volatile compounds such as ethyl acetate that smells like fingernail polish. High VA can also lead to stuck alcoholic fermentations. The legal limits for VA in red and white table wines in the U.S. are 0.12% (g/100 ml) and 0.11% (g/100 ml), respectively. The VA concentration in wines for export may not exceed 0.09% (g/100 ml) (http://waterhouse.ucdavis.edu/winecomp/va.htm). In Australia, the maximum VA allowed is 0.15% (g/100 ml) (as acetic acid) (http://www.foodstandards.gov.au/foodstandards/foodstandardscode.cfm). In the European Community, the maximum limit for red wines is 0.12% (g/100 ml) and 0.108% (g/100 ml) for white wines (http://www.awbc.com.au/exporting/exportgrid/index.asp). Botrytis infected wines may contain a maximum level of 0.15% (g/100 ml). Once the VA exceeds the legal limit, wines have to be blended with wine containing lower amounts of acetic acid, which leads to a reduction in the quality of wine. Therefore, the ability to regulate acetic acid levels is a critical tool for controlling wine quality. Here we have shown that a transcription factor, Yml081Wp, controls acetaldehyde dehydrogenase by regulating the expression of the genes, particularly *ALD6*, that encode this activity. As a consequence, the conversion of acetaldehyde to acetate and the level of acetic acid in the media can be controlled. We propose renaming the YML081W ORF as *AAF1*, for Acetic Acid Factor.

## Supporting Information

Table S1 **Oligonucleotide primer list.** Sequences of primers used for the amplification of DNA cassettes by PCR are listed, along with the DNA template, and the purpose of each PCR reaction. The amplification products were either used directly for transformation into yeast cells, or cloning into vectors.
(DOC)

Table S2 ***S. cerevisiae*** **strains used in these experiments.** A listing of the genotypes of each strain described in the text of this paper.
(DOC)
