# Supplementary material for: The Fermentation Stress Response Protein Aaf1p/Yml081Wp Regulates Acetate Production in Saccharomyces cerevisiae
Source: PLoS One. 2012 Dec 11;7(12):e51551. doi: 10.1371/journal.pone.0051551 (PMC3519862; doi:10.1371/journal.pone.0051551)
Supplement: Table S1 — Oligonucleotide primer list. Sequences of primers used for the amplification of DNA cassettes by PCR are listed, along with the DNA template, and the purpose of each PCR reaction. The amplification products were either used directly for transformation into yeast cells, or cloning into vectors. (DOC) [file pone.0051551.s001.doc]

**Table S1: Oligonucleotide primer list**

| sequence | template | purpose |
| --- | --- | --- |
| cccagttgtcttctgttctatcagcagcgaatatttcagcttcttgtaattgtacgttgcatctgccatgagctgaagcttcgtacg | pUG6 | construction of YML081WΔ strain |
| tttttcaatttgccctaaagaactaatataatgttacatacggatatgctaaatatctatctaaagtctatacgactcactataggg | pUG6 | construction of YML081WΔ strain |
| atagacgatagcactttggg | M2 genomic DNA | check YML081W genotype |
| tgttaccaatcaagcgctgg | M2 genomic DNA | check YML081W genotype |
| attaagggttgtcgacccttaatttttattttagattcc | pHVX2 | construction of pCW1 |
| tacgctgcaggtcgatgttttatatttgttgtaaaaag | pHVX2 | construction of pCW1 |
| gtcttctgttctatcagcagcgaatatttcagcttcttgtaattgtacgttgcatctgccttaatacgactcactataggg | pCW1 | construction of YML081W overexpression strain |
| agcatagatagtggaggatatatctcgtttgatcggaagtcctttgaattcttccgatgacattgttttatatttgttgtaaaaagtag | pCW1 | construction of YML081W overexpression strain |
| aaagggtttctcgttcgtatgtgc | M2 genomic DNA | check *PGK1* promoter-YML081W genotype |
| tcaaacagaattgtccgaatcg | M2 genomic DNA | check *PGK1* promoter-YML081W genotype |
| ggtaactacgcgagcaacttctattaagagaaataatttttgggaaatggcctgtttcggagctgaagcttcgtacgc | pUG6 | construction of *RSF2*Δ strain |
| tctaagctttaattctgtaaatactatagtatagagacggccgccattatatatttgtaa taggccactagtggatctg | pUG6 | construction of *RSF2*Δ strain |
| atttcttaagatggcaggac | M2 genomic DNA | check *RSF2* genotype |
| tgctttcttcattgtcatcg | M2 genomic DNA | check *RSF2* genotype |
| aggattagaagtatctggaaaaccaaccaagaaaactacaataacaaaaataaataaagcagctgaagcttcgtacgc | pUG6 | construction of *ALD4*Δ strain |
| gacagaatatttaattttatgtatgtaagcatcgattggacaccaggcttattgatgacctaggccactagtggatctg | pUG6 | construction of *ALD4*Δ strain |
| agccaactgtctttggtgac | M2 genomic DNA | check *ALD4* genotype |
| aagtttcatcaaggtctctg | M2 genomic DNA | check *ALD4* genotype |
| tagaagaaaaaacatcaagaaacatctttaacatacacaaacacatactatcagaatacaagctgaagcttcgtacgc | pUG6 | construction of *ALD6*Δ strain |
| gtaagaccaagtaagtttatatgaaagtattttgtgtatatgacggaaagaaatgcaggttaggccactagtggatctg | pUG6 | construction of *ALD6*Δ strain |
| accaaccgttttctacgatg | M2 genomic DNA | check *ALD6* genotype |
| tagcagttgttgtacactag | M2 genomic DNA | check *ALD6* genotype |
| ttttggcgttgttgctcaaa | M2 cDNA | *ALD2* qPCR |
| accgtgttaccggctgctaa | M2 cDNA | *ALD2* qPCR |
| cctggttatggttccgttgtg | M2 cDNA | *ALD3* qPCR |
| caatactgagccgccaacct | M2 cDNA | *ALD3* qPCR |
| tacgggttggctgctggtat | M2 cDNA | *ALD4* qPCR |
| actgcgtggtggaaatcgtt | M2 cDNA | *ALD4* qPCR |
| ttgcagcggctaaaaaagct | M2 cDNA | *ALD5* qPCR |
| ggcagccagtgtttcttggt | M2 cDNA | *ALD5* qPCR |
| gctgcctatgccgacaaagt | M2 cDNA | *ALD6* qPCR |
| tgaccacagacaccgattgg | M2 cDNA | *ALD6* qPCR |
| aagcgatttccatgcgaaga | M2 cDNA | YML081W qPCR |
| tggcaggttttcgtcactga | M2 cDNA | YML081W qPCR |
| tctgttgatgctttacaaaactacttgcaagttaaagcggtccgtgccaaattggacgagagggaacaaaagctggag | p3FLAG-KanMX | construction of FLAG-tagged *ALD4* |
| gacagaatatttaattttatgtatgtaagcatcgattggacaccaggcttattgatgaccctatagggcgaattgggt | p3FLAG-KanMX | construction of FLAG-tagged *ALD4* |
| gaaatgggtgaagaagtctaccatgcatacactgaagtaaaagctgtcagaattaagttgagggaacaaaagctggag | p3FLAG-KanMX | construction of FLAG-tagged *ALD6* |
| caagtaagtttatatgaaagtattttgtgtatatgacggaaagaaatgcaggttggtacactatagggcgaattgggt | p3FLAG-KanMX | construction of FLAG-tagged *ALD6* |
| ccgcgtacgcgtcgacctgttgcccgtctcactg | pMELβ2 | construction of pCW5 |
| attcggtaccgtcgaaaaactgtattataagtaaatgcatgtatactaaactcac | pMELβ2 | construction of pCW5 |
| cgggcaacaggtcgatgtcaaagtgtagcttagtcattgtattctg | M2 genomic DNA | construction of pCW5 *ALD6* promoter-*lacZ* reporter (+22) |
| ccgcgtacgcgtcgaacaactttccgcggacgc | M2 genomic DNA | construction of pCW5 *ALD6* promoter-*lacZ* reporter (-782) |
| ccgcgtacgcgtcgatcgccgtggctgatgagg | M2 genomic DNA | construction of pCW5 *ALD6* promoter-*lacZ* reporter (-636) |
| ccgcgtacgcgtcgacagtggcctgtttttcgac | M2 genomic DNA | construction of pCW5 *ALD6* promoter-*lacZ* reporter (-586) |
| ccgcgtacgcgtcgacgtatccaagccgaaacgg | M2 genomic DNA | construction of pCW5 *ALD6* promoter-*lacZ* reporter (-520) |
| ccgcgtacgcgtcgaccgcggacgtgtaaaaagatatgc | M2 genomic DNA | construction of pCW5 *ALD6* promoter-*lacZ* reporter (-414) |
| gaggtctcctcttgcccgggccgcagaaaaatatatcagtggcctgtttttcg | pCW5 *ALD6* promoter -636-*lacZ* reporter | Mutation of potential YML081W binding sites |
| cgaaaaacaggccactgatatatttttctgcggcccgggcaagaggagacctc | pCW5 *ALD6* promoter -636-*lacZ* reporter | Mutation of potential YML081W binding sites |
| tgctgaaaggcgattcagtcg | pCW5 *lacZ* reporters | Sequencing of pCW5 *lacZ* reporters |
