# Supplementary material for: The Fermentation Stress Response Protein Aaf1p/Yml081Wp Regulates Acetate Production in Saccharomyces cerevisiae
Source: PLoS One. 2012 Dec 11;7(12):e51551. doi: 10.1371/journal.pone.0051551 (PMC3519862; doi:10.1371/journal.pone.0051551)
Supplement: Table S2 — S. cerevisiae strains used in these experiments. A listing of the genotypes of each strain described in the text of this paper. (DOC) [file pone.0051551.s002.doc]

**Table S2: *S. cerevisiae* strains used in these experiments**

| name | genotype |
| --- | --- |
| M2 | *MATα*/*MATa* |
| *yml081wΔ* | M2, *yml081wΔ::kanMX4*/*yml081wΔ::kanMX4* |
| p*PGK1*-YML081W | M2, *kanMX4-*p*PGK1*-YML081W/*kanMX4-*p*PGK1-*YML081W |
| *rsf2Δ* | M2, *rsf2Δ::kanMX4*/*rsf2Δ::kanMX4* |
| *ALD4-*FLAG | M2, *ALD4-*FLAG-*natMX*/*ALD4-*FLAG-*natMX* |
| *ALD4-*FLAG/*yml081wΔ* | M2*,* *ALD4-*FLAG-*natMX*/*ALD4-*FLAG-*natMX yml081wΔ::kanMX4*/*yml081wΔ::kanMX4* |
| *ALD4-*FLAG/p*PGK1-*YML081W | M2*,* *ALD4-*FLAG-*natMX*/*ALD4-*FLAG-*natMX kanMX4-*p*PGK1-*YML081W/*kanMX4-*p*PGK1-*YML081W |
| *ALD6-*FLAG | M2, *ALD6-*FLAG-*natMX*/*ALD6-*FLAG-*natMX* |
| *ALD6-*FLAG/*yml081wΔ* | M2, *ALD6-*FLAG-*natMX*/*ALD6-*FLAG-*natMX yml081wΔ::kanMX4*/*yml081wΔ::kanMX4* |
| *ALD6-*FLAG/p*PGK1-*YML081W | M2, *ALD6-*FLAG-*natMX*/*ALD6-*FLAG-*natMX kanMX4-*p*PGK1-*YML081W/*kanMX4-*p*PGK1-*YML081W |
| *ald4Δ* | M2, *ald4Δ::hphMX4*/*ald4Δ::hphMX4* |
| *ald4Δ/yml081wΔ* | M2, *ald4Δ::hphMX4*/*ald4Δ::hphMX4 yml081wΔ::kanMX4*/*yml081wΔ::kanMX4* |
| *ald6Δ* | M2, *ald6Δ::hphMX4/ald6Δ::hphMX4* |
| *ald6Δ/yml081wΔ* | M2, *ald6Δ::hphMX4/ald6Δ::hphMX4* *yml081wΔ::kanMX4*/*yml081wΔ::kanMX4* |
